# SARS-CoV-2 infection in an infant with severe dilated cardiomyopathy

**DOI:** 10.1017/S1047951120004060

**Published:** 2020-10-29

**Authors:** Aslak Widerøe Kristoffersen, Per Kristian Knudsen, Thomas Møller

**Affiliations:** 1 Department of Paediatric Cardiology, Division of Paediatric and Adolescent Medicine, Oslo University Hospital, Oslo, Norway; 2 Department of Paediatric Medicine, Division of Paediatric and Adolescent Medicine, Oslo University Hospital, Oslo, Norway

**Keywords:** COVID-19, SARS-CoV-2, dilated cardiomyopathy, infant, heart failure

## Abstract

A four- and a half-month-old girl with severe dilated cardiomyopathy due to neonatal enterovirus myocarditis, treated with diuretics and milrinone for the past 4 months, was infected with SARS-CoV-2. The disease course was characterised by high fever and gastrointestinal symptoms. Cardiac function, as measured by echocardiography, remained stable. The treatment focused on maintaining a normal heart rate and a stable fluid balance. In children with severe underlying cardiac disease, even a mild SARS-CoV-2 infection can require close monitoring and compound treatment.

## Background

Although children have been affected less severely by the COVID-19 pandemic than adults^1^, a concern has been raised regarding the risk of severe COVID-19 in the paediatric population with chronic illnesses, such as severe heart disease^2^.

**Figure 1.**
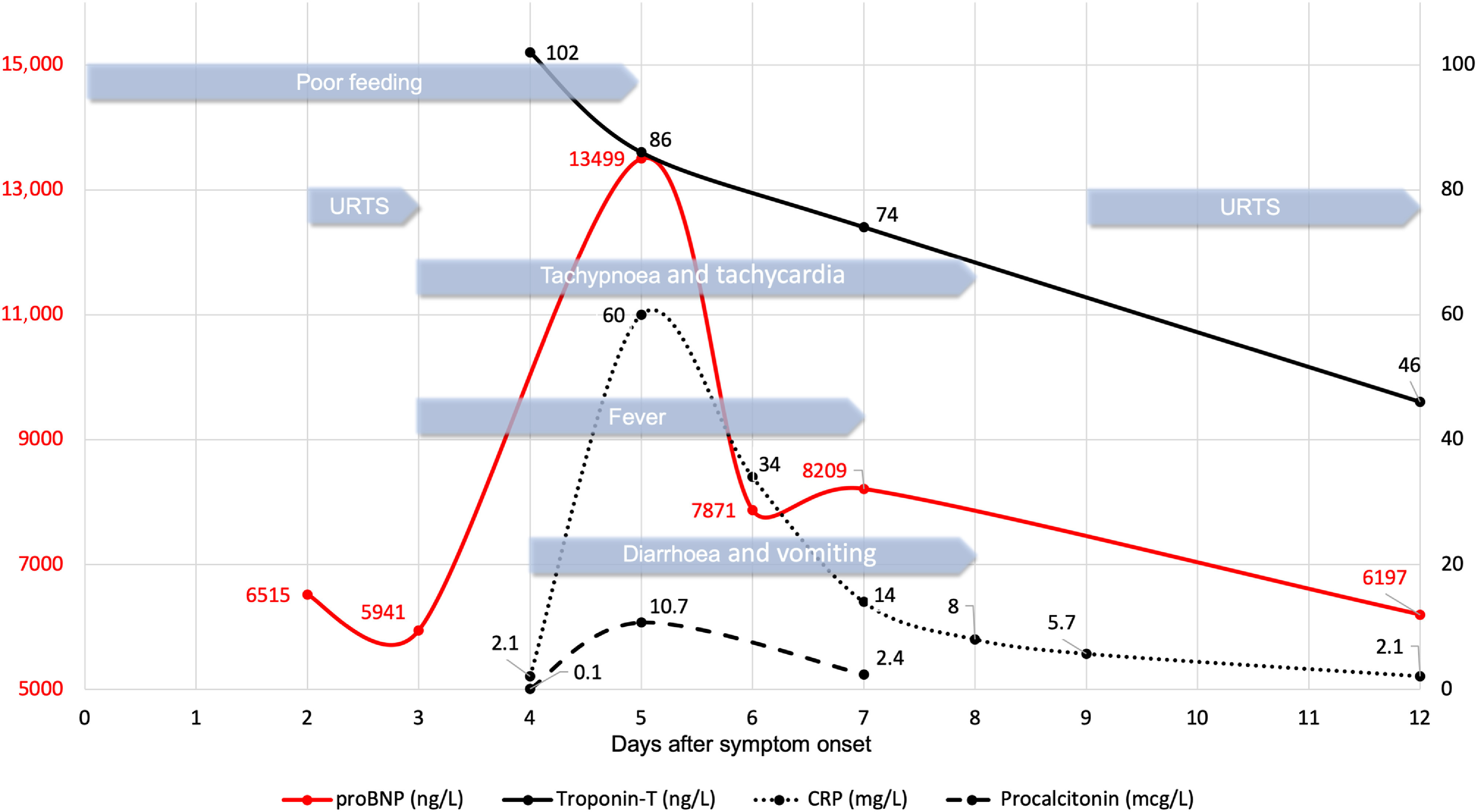
Timeline of clinical features and laboratory results during the course of SARS-CoV-2 infection in a 4.5-month-old infant with dilated cardiomyopathy. URTS = upper respiratory tract symptoms.

## Case report

We report a case of COVID-19 in a four- and a half-month-old girl with dilated cardiomyopathy due to neonatal enterovirus myocarditis. She had been treated with diuretics and milrinone for the past 4 months, and serial echocardiographic studies had shown an increasingly dilated left ventricle with severely reduced left ventricular systolic function and preserved right ventricular function (left ventricular end-diastolic diameter z score 3–7, global longitudinal strain −7%, left ventricle biplane ejection fraction 17–25%, and right ventricle fraction area change 50–60%). At 4 months of age, N-terminal pro-brain natriuretic peptide (pro-BNP) and troponin T stabilised at approximately 6000 and 100 ng/L, respectively.

After completing a pretransplant diagnostic work up, our patient started feeding poorly at four and a half months of age (Fig 1). Three days later, she developed mild rhinorrhoea and cough. On the morning of day 4, she became febrile with a body temperature of 40.5 °C The doctor on call reported a pale but alert infant. Her respiratory rate was 60 breaths per minute with an oxygen saturation of 98% on ambient air. She did not have dyspnoea, chest retractions, or wheezing. Lung auscultation was normal. Her pulse rate was 150–200 beats per minute, capillary refill time was less than 3 seconds, and blood pressure was 88/33 (51) mmHg. Heart auscultation was normal, and the physical examination was otherwise unremarkable.

From days 4 to 7, the clinical condition was characterised by frequent loose stools, abdominal discomfort, and fluctuating fever accompanied by tachycardia and tachypnea, but without the need for supplemental oxygen or other signs of respiratory distress. Starting on day 8, she no longer had a fever, and by day 10, she was back to her habitual state only, with mild hoarseness lasting a few more days. Her oxygen saturation was >95% without oxygen supplementation, and her blood pressure was within the normal range for age during the entire disease period.

A nasopharyngeal swab specimen taken on day 4 came back positive for SARS-CoV-2 RNA. All other tests for airway pathogens were negative (influenza virus A and B, parainfluenza virus 1–4, respiratory syncytial virus, rhinovirus, adenovirus, enterovirus, metapneumovirus, *Mycoplasma pneumonia*, and *Chlamydophila pneumoniae*). On day 5, additional microbiological tests were performed, and all came back negative or showed no signs of active infection: blood and urinary cultures, tests for faecal pathogens, serologic tests for cytomegalovirus, Epstein–Barr virus and parvovirus B19, and PCR analyses in blood for enterovirus, parechovirus, and adenovirus. Two days after recovery, 12 days after the onset of symptoms, a faecal sample was positive for SARS-CoV-2 RNA and negative for enterovirus and parechovirus.

Laboratory examinations showed serum C-reactive protein and procalcitonin concentrations reaching maximum levels of 60 mg/L and 10.7 mcg/L, respectively, on day 5. Her pro-BNP peaked at 13,499 ng/L on day 5 and then declined. Her troponin T was 102 ng/L on day 4, falling to 74 ng/L on day 7. Creatinine, carbamide, ferritin, lactate dehydrogenase, alanine aminotransferase, and aspartate aminotransferase were within normal ranges during the entire course of illness. Except for mild leukopenia (leukocyte count 3.6 × 10^9^/L on day 9) and granulocytopenia (neutrophil count 0.6 × 10^9^/L on day 8), the other haematologic parameters stayed within the normal range. Screening laboratory tests for immunodeficiency (level of immunoglobulins and T- and B-cell subpopulations) were normal.

Cardiac ultrasound was performed on days 5, 7, and 12. The measurements stayed stable during the disease course (global longitudinal strain −6%, left ventricular end-diastolic diameter 39 mm (z score 5), and left ventricle biplane ejection fraction 19%).

A chest radiograph taken on day 4 showed cardiomegaly and pulmonary venous congestion as before. However, the second chest radiograph on day 8 showed centrally located opacities consistent with worsened congestion. On a follow-up chest radiograph acquired 3 weeks later, the opacities had regressed.

During the febrile period, our patient was put on regular antipyretics (acetaminophen and ibuprofen). When diarrhoea started on day 5, nasogastric feeding was increased by 15–20% to compensate for her losses, and diuretics were given intravenously instead of orally to overcome the impaired gastrointestinal absorption. She was treated with antibiotics (cefotaxime) from days 5 to 7 because a bacterial infection could not be excluded. Because of long-standing episodes of crying and discomfort accompanied by sinus tachycardia up to 200 beats per minute, she was put on a fixed dosage of clonidine combined with morphine as needed starting on day 5 to lower her heart rate. Our patient did not need respiratory support or oxygen supplementation at any point in time.

After recovery, her pro-BNP level has been between 2000 and 7000 ng/L until now (3 months post-infection). Cardiac ultrasound shows no signs of improvement. According to a consensus in our institution’s heart transplant conference, the patient was listed for heart transplantation 1 week after the cessation of all COVID-19 symptoms.

## Discussion

The course of COVID-19 in children has been reported to be mostly mild^1,3,4^. Comorbidities have been reported in children with COVID-19 requiring hospitalisation and even respiratory support^5,6^. However, so far, we have limited knowledge about the clinical presentation and disease severity in children with congenital or acquired heart disease infected with SARS-CoV-2^2^.

Our patient had severe heart failure compensated and stabilised by continuous i.v. inotropic/vasodilating treatment. During that stage, even a mild infection had to be considered a serious and potentially life-threatening event.

During the course of COVID-19, high fever and frequent loose stools created a challenge for maintaining fluid balance. Moreover, tachycardia as a result of fever, pain, and increased metabolic demands could have had devastating effects. Strict fluid balance, antipyretics, pain relief, and light sedation were sufficient to support our patient through the infection.

To the best of our knowledge, this is the first reported case of COVID-19 in an infant with end-stage heart failure. The disease caused fever and gastrointestinal symptoms but only mild respiratory symptoms. There were no signs of major inflammatory, cardiac, or pulmonary involvement from the virus. Nonetheless, our case illustrates that in children with severe underlying disease, even a mild SARS-CoV-2 infection may cause constitutional symptoms that require compound treatment measures to be taken.
